# Function of L-Pipecolic Acid as Compatible Solute in *Corynebacterium glutamicum* as Basis for Its Production Under Hyperosmolar Conditions

**DOI:** 10.3389/fmicb.2019.00340

**Published:** 2019-02-25

**Authors:** Fernando Pérez-García, Luciana F. Brito, Volker F. Wendisch

**Affiliations:** Genetics of Prokaryotes, Faculty of Biology – CeBiTec, Bielefeld University, Bielefeld, Germany

**Keywords:** pipecolic acid, osmo regulation, compatible solute, proline, *Corynebacterium glutamicum*, RNAseq analysis, solute export, solute uptake

## Abstract

Pipecolic acid or L-PA is a cyclic amino acid derived from L-lysine which has gained interest in the recent years within the pharmaceutical and chemical industries. L-PA can be produced efficiently using recombinant *Corynebacterium glutamicum* strains by expanding the natural L-lysine biosynthetic pathway. L-PA is a six-membered ring homolog of the five-membered ring amino acid L-proline, which serves as compatible solute in *C. glutamicum*.

Here, we show that *de novo* synthesized or externally added L-PA partially is beneficial for growth under hyper-osmotic stress conditions. *C. glutamicum* cells accumulated L-PA under elevated osmotic pressure and released it after an osmotic down shock. In the absence of the mechanosensitive channel YggB intracellular L-PA concentrations increased and its release after osmotic down shock was slower. The proline permease ProP was identified as a candidate L-PA uptake system since RNAseq analysis revealed increased *proP* RNA levels upon L-PA production. Under hyper-osmotic conditions, a Δ*proP* strain showed similar growth behavior than the parent strain when L-proline was added externally. By contrast, the growth impairment of the Δ*proP* strain under hyper-osmotic conditions could not be alleviated by addition of L-PA unless *proP* was expressed from a plasmid. This is commensurate with the view that L-proline can be imported into the *C. glutamicum* cell by ProP and other transporters such as EctP and PutP, while ProP appears of major importance for L-PA uptake under hyper-osmotic stress conditions.

## Introduction

In nature, all living organisms must respond to environmental fluctuations to survive. For example, bacteria have developed defense mechanisms for hyper- and hypo-osmotic external conditions to maintain cell viability including the *de novo* synthesis or uptake of osmo compatible solutes such as betaines. Since plants also contain these osmo compatible solutes, they are commonly present in production media and, thus, relevant for biotechnological fermentations (Farwick et al., 1995). The industrial workhorse *Corynebacterium glutamicum* disposes of mechanosensitive channels (MSCs) which perform as emergency release valves (Ruffert et al., 1999). After an osmotic downshift and to avoid cell lysis, MSCs are immediately activated by membrane turgor pressure to release solutes and to decrease in the internal osmolality (Ruffert et al., 1997; Morbach and Krämer, 2003). *C. glutamicum* can synthesize proline, glutamine, and trehalose and use them as compatible solutes, whereas it cannot synthesize ectoine and betaine, which therefore only function as compatible solutes when present in the environment (Frings et al., 1993; Farwick et al., 1995; Guillouet and Engasser, 1995; Wolf et al., 2003). Proline is the major *de novo* synthetized compatible solute in *C. glutamicum* (Skjerdal et al., 1996; Wolf et al., 2003). *De novo* biosynthesis of proline is induced under osmostress-conditions (Rönsch et al., 2003) unless nitrogen is scarce, a condition when trehalose is synthesized instead of proline (Wolf et al., 2003). Externally added proline can be taken up into the *C. glutamicum* cell by the import systems EctP, ProP, and PutP (Peter et al., 1998). The import of proline by the carriers EctP and ProP is osmoregulated (Peter et al., 1998), while the import of proline by PutP is not (Peter et al., 1997).

The cyclic amino acid L-pipecolic (L-PA), also known as homoproline, is a non-proteogenic amino acid and an intermediate of the catabolism of D,L-lysine (Neshich et al., 2013). L-PA is similar in chemical structure to L-proline since they only differ in ring size by one carbon (Pérez-García et al., 2016). L-PA plays many roles in microorganisms, plants, and animals; including the interactions between organisms and as precursor of natural bioactive molecules (Vranova et al., 2013). Notably, L-PA was reported as compatible solute for the microorganisms *Silicibacter pomeroyi, Sinorhizobium meliloti*, and *Escherichia coli* (Gouesbet et al., 1994; Gouffi et al., 2000; Neshich et al., 2013). *E. coli* does not degrade lysine to L-PA, but to cadaverine by the lysine decarboxylases LdcC or CadA (Mimitsuka et al., 2007; Kind et al., 2010). However, externally added L-PA protected *E. coli* cells under high osmolarity conditions (Gouesbet et al., 1994). *C. glutamicum* lacks lysine catabolic pathways, although the production of L-lysine-derived compounds has been established in *C. glutamicum* by metabolic engineering (Kind et al., 2010; Pérez-García et al., 2016; Jorge et al., 2017). A lysine producing *C. glutamicum* strain was engineered to overproduce L-PA by heterologous expression of the lysine dehydrogenase gene (*lysDH*) from *S. pomeroyi* and overexpression of the native pyrroline-5-carboxylate reductase gene (*proC*) from *C. glutamicum* (Pérez-García et al., 2016). However, the physiological role of L-PA for *C. glutamicum* has not yet been described. Here, we characterized the effect of L-PA on *C. glutamicum* (either added to the culture medium or synthesized *de novo*) under different conditions of osmotic pressure by physiological and RNAseq experiments. We provide evidence that YggB may be involved in export of L-PA and ProP in its import into the *C. glutamicum* cell.

## Materials and Methods

### Strain, Plasmids, and Culture Conditions

The strains and plasmids used in this work are listed in Table 1. *E. coli* strains were routinely cultivated in LB medium (10 g tryptone, 5 g yeast extract and 10 g NaCl per liter) or on LB agar plates at 37°C. *C. glutamicum* strains were routinely precultivated in brain heart infusion (BHI, ROTH^®^) plates or liquid medium overnight at 30°C. For *C. glutamicum* main cultures in flask, CGXII medium (Eggeling and Bott, 2005) was inoculated to an initial OD_600_ of 1 using 4% (w/v) glucose as sole-carbon source. For *C. glutamicum* main cultures in BioLector (m2p-labs, Baesweiler, Germany), growth experiments were performed in Flowerplates at 1,000 rpm, 95% humidity, 30°C and backscatter gain 15, inoculated to an initial OD_600_ of 1 and using 4% (w/v) glucose as sole-carbon source. When necessary, the growth medium was supplemented with kanamycin (25 μg mL^-1^), spectinomycin (100 μg mL^-1^) and isopropyl β-D-1-thiogalactopyranoside (IPTG) (1 mM). For growth in hyperosmolar conditions 100, 200, or 400 mM of NaCl were added to the medium.

**Table 1:** Strains and plasmids used in this work.

Strains and plasmids	Description	Source
Strains		
GSL	*C. glutamicum* ATCC13032 with the following modifications: Δ*pck, ΔsugR, ΔldhA, pyc^P458S^, hom^V 59A^*, two copies of *lysC^T311I^*, two copies of *asd*, two copies of *dapA*, two copies of *dapB*, two copies of *ddh*, two copies of *lysA*, two copies of *lysE*, in-frame deletion of prophages CGP1 (cg1507-cg1524), CGP2 (cg1746-cg1752), and CGP3 (cg1890-cg2071)	Pérez-García et al., 2016
GSLΔ*yggB*	In-frame deletion of *yggB* (cg1434) in GSL	This work
GSLΔ*proP*	In-frame deletion of *proP* (cg3395) in GSL	This work
JJ001	*C. glutamicum* ATCC13032 with the following modifications:Δ*argF, ΔargR* (auxotrophic for L-arginine); carrying the vector pVWEx1	Jensen and Wendisch, 2013
JJ004	JJ001 strain carrying the vector pVWEx1-ocdPp(TAA)	Jensen and Wendisch, 2013
*E. coli* DH5α	F^-^*thi*-1 *endA*1 *hsdr*17 (r^-^, m^-^) *supE*44 Δ*lacU*169 (Φ80*lacZ*ΔM15) *recA*1 *gyrA*96 *relA*1	Hanahan, 1983
*E. coli* S17-1	*recA, thi, pro, hsd* R-M+ (RP4: 2-Tc:Mu-:Km, integrated into the chromosome)	Simon et al., 1983
Plasmids		
pVWEx1	Km^R^, *C. glutamicum*/E. coli shuttle vector (Ptac, *lacI*, pHM1519 *oriV*_Cg_)	Peters-Wendisch et al., 2001
pEKEx3	Spec^R^, *C. glutamicum*/*E. coli* shuttle vector (Ptac, *lacI*, pBL1 *oriV*_Cg_)	Stansen et al., 2005
pVWEx1-*lysDH-proC*	Km^R^, pVWEx1 overexpressing *lysDH* from *S. pomeroyi* DSS-3 and *proC* from *C. glutamicum* ATCC 13032	Pérez-García et al., 2016
pEKEx3-*yggB*	Spe^R^, pEKEx3 overexpressing *yggB* from *C. glutamicum* ATCC 13032	This work
pEKEx3-*proP*	Spe^R^, pEKEx3 overexpressing *proP* from *C. glutamicum* ATCC 13032	This work
pK19*mobsacB*	Km^R^; *E. coli*/*C. glutamicum* shuttle vector for construction of insertion and deletion mutants in *C. glutamicum* (pK18 *oriVEc sacB lacZα*)	Schaffer et al., 2001
pK19*mobsacB*-Δ*yggB*	pK19*mobsacB* with a *yggB* (cg1434) deletion construct	Lubitz and Wendisch, 2016
pK19*mobsacB*-Δ*proP*	pK19*mobsacB* with a *proP* (cg3395) deletion construct	This work


### Molecular Biology Methods

As host for gene cloning *E. coli* DH5α was used (Hanahan, 1983). *E. coli* was transformed by heat shock following the method described elsewhere (Hanahan, 1983), while *C. glutamicum* was transformed by electroporation following the method described elsewhere (Eggeling and Bott, 2005). The pair of primers YgFw/YgRv (Table 2) were used to amplified *yggB* from genomic DNA of *C. glutamicum* ATCC 13032. The pair of primers PrFw/PrRv (Table 2) were used to amplified *proP* from genomic DNA of *C. glutamicum* ATCC 13032. The amplified genes were cloned by Gibson assembly (Gibson, 2011) into the vector pEKEx3 (Stansen et al., 2005) digested with BamHI, yielding the vectors pEKEx3-*yggB* and pEKEx3-*proP*. Positive clones were verified by colony PCR using the pair of primers X1Fw/X1Rv (Table 2). The up- and downstream regions of *proP* gene were amplified by PCR from genomic DNA of *C. glutamicum* ATCC 13032 using the pair of primers PrDA/PrDB and PrDC/PrDD. The up and down amplified fragments were fused by cross-over PCR with primer pair PrDA/PrDD and cloned by ligation (Eggeling and Bott, 2005) into the vector pK19*mobsacB* (Schäfer et al., 1994) restricted with BamHI. Positive clones were verified by colony PCR using the pair of primers 196F/197R (Table 2). The resulting vector pK19mobsacB-*gdh* was transferred to *E. coli* S17-1. In-frame deletion of the *yggB* and *proP* genes from *C. glutamicum* was performed via a two-step homologous recombination method (Eggeling and Bott, 2005). The pK19*mobsacB* vectors were transferred to the GSL strain via conjugation using *E. coli* S17-1 (Simon et al., 1983). The deletions of *yggB* and *proP* were verified by colony PCR using the pair of primers DE31/DE32 and PrDE/PrDF, respectively.

**Table 2:** Oligonucleotide sequences used in this work for molecular cloning and in-frame deletion.

Primer	Sequence (5→3)
PrFw	GCATGCCTGCAGGTCGACTCTAGAGGAAAGGAGGCCCTTCAGGTGAGCCCGATTCGCTC
PrRv	AATTCGAGCTCGGTACCCGGGGATCTTATGCGTTTTGCTTTTCAG
YgFw	GCATGCCTGCAGGTCGACTCTAGAGGAAAGGAGGCCCTTCAGATGATTTTAGGCGTACCC
YgRv	AATTCGAGCTCGGTACCCGGGGATCCTAAGGGGTGGACGTCGG
PrDA	GCATGCCTGCAGGTCGACTCTAGAGTTCGGTGCCCTCCACGGCAC
PrDB	GGGTAGGTGATTTGAATTTGTGAGTAAAACCTCTCGTCATATC
PrDC	ACAAATTCAAATCACCTACCCCCGTAAAGCCCGCTGCAAGG
PrDD	AATTCGAGCTCGGTACCCGGGGATCGTAACGATGCAGACCGCCGG
PrDE	CGGTGCCCTCCACGGCACC
PrDF	AACGATGCAGACCGCCGGCG
DE31	CTTTTGGCGCTCCAAGTACT
DE32	TCCTCGAGCGATCGAACAAT
X1Fw	CATCATAACGGTTCTGGC
X1Rv	ATCTTCTCTCATCCGCCA
196F	CGCCAGGGTTTTCCCAGTCACGAC
197R	AGCGGATAACAATTTCACACAGGA


### Internal Amino Acids Extraction

For the quantification of intracellular L-PA 2 mL of liquid medium were collected. One milliliter was centrifuged at 14,000 rpm and 4°C for 10–15 min. The resulting pellets were resuspended and treated with 5% HClO_4_ in an ice bath for 30 min. Then, the supernatant was neutralized with K_2_CO_3_ solution and centrifuged again at 14,000 rpm and 4°C for 10–15 min. Afterward, the supernatants were directly used for L-PA quantification or stored at -20°C (Sun et al., 2016). It has to be noted that the water space of the pellet in such a centrifugation step will contain compounds presents in the extracellular volume and this will affect the determination of the intracellular concentration. On the other hand, intracellular compounds may leak out of the cell during washing steps and this will also affect the determination of the intracellular concentration. Since all samples were processed in the same way, the possible fluctuations/errors in the measurements should affect all samples similarly. Exact quantitation would require methods such as described by Klingenberg and Pfaff (1967) which combine centrifugation through silicone oil for fast separation of supernatant and pellet by centrifugation and inactivation of the pellet by perchloric acid. The second collected mL was used to determine the biomass according to the correlation CDW [g L^-1^] = 0.35 OD (Bolten et al., 2007).

### Determination of L-PA by High Pressure Liquid Chromatography

The concentration of L-PA was quantified by using high-pressure liquid chromatography. The samples from the cell cultures were collected by centrifugation (14,000 rpm, 15 min and at room temperature), and further used for analysis. The samples were derivatized with fluorenylmethyl chloroformate (FMOC) as described (Schneider and Wendisch, 2010). Amino acid separation was performed on a system consisting of a pre-column (LiChrospher 100 RP18 EC-5μ (40 × 4 mm), CS-Chromatographie Service GmbH, Langerwehe, Germany) and a main column (LiChrospher 100 RP18 EC-5μ (125 × 4 mm), CS Chromatographie Service GmbH). The detection was carried out with a fluorescence detector with the excitation and emission wavelength of 230 nm and 310 nm, respectively (FLD G1321A, 1200 series, Agilent Technologies).

### RNAseq Analysis

For extraction of *C. glutamicum* bacterial cell pellets grown under the experimental conditions were harvested at mid-exponential phase. Harvesting procedure was done according to Irla et al. (2015) and cell pellets were kept at -80°C for further RNA isolation. Then, the pellets were thawed in ice and RNA was isolated individually for each sample using NucleoSpin RNA isolation kit (Macherey-Nagel, Düren, Germany). RNA samples with genomic DNA contamination were treated with the RNase-free DNase set (Qiagen, Hilden, Germany) (Brito et al., 2017). The concentration of isolated RNA was determined by DropSense^TM^ 16 (Trinean, Ghent, Belgium; software version 2.1.0.18). To verify the quality of RNA samples, we performed capillary gel electrophoresis (Agilent Bioanalyzer 2100 system using the Agilent RNA 6000 Pico kit; Agilent Technologies, Böblingen, Germany). The extracted RNA samples were pooled in equal parts and the pool of total RNA was subsequently used for the preparation of the cDNA libraries. The preparation and sequencing of the libraries were performed as described elsewhere (Mentz et al., 2013; Irla et al., 2015). Then, the reads were trimmed to a minimal length of 20 base pairs and in paired end mode with the Trimmotatic ver. 0.33 (Bolger et al., 2014). Trimmed reads were mapped to the reference genome of *C. glutamicum* ATCC13032 (Kalinowski et al., 2003) using the software Bowtie (Langmead et al., 2009). In order to perform differential gene expression analysis (DEseq) (Anders and Huber, 2010), we used the software for visualization of mapped sequences ReadXplorer (Hilker et al., 2014).

### Real-Time Quantitative Reverse Transcription-PCR

The real-time quantitative reverse transcription-PCR (qRT-PCR) was performed in order to validate the data obtained by DEseq analysis by using the CFX Connect^TM^ Real-Time PCR Detection System (Bio-Rad Laboratories, Irvine, CA, United States). Same RNA samples utilized in the RNAseq analysis were utilized as templates for qRT-PCR. All samples RNA concentration was adjusted to 50 ng μL^-1^. Afterward, 1 μL for each sample was pipetted into a reaction mix of the SensiFAST^TM^ SYBR^®^ No-ROX Kit (Bioline, Luckenwalde, Germany), following manufacturer’s instructions. Differentially expressed genes in DEseq analysis were selected as targets for qRT-PCR amplifications (primers listed in Table 3). The melting-curve data-based quantification cycle (Cq) values, from the LightCycler^®^ output files, were used for further calculation as it is described elsewhere (Crooks et al., 2004).

**Table 3:** Oligonucleotide sequences (5→3) used for amplification of gene fragments in qRT-PCR.

Gene identity	Forward	Reverse	Gene product length (bp)
*betP*	GCGGGCTTGCTTGAGAATCC	TGAAGGCCCAGCCGAGATTG	232
cg0569	AGCTTTGGCTGCTTCAGTAG	AGATTCCATGCCGGAACTTG	241
cg1665	GCTGCCAACTCTGCAACCTC	CCATTCGGGCCTTCTTCCAC	245
cg2677	GGCTCTGCCTCCATTCTTTG	GGTTGTGCCTTGACCTCTTC	210
cg2851	CAACGTGAACACGGTGTATC	CACATCGTCGAATCCGTTTG	210
cg3254	ATGCTTGCCCTAGGTTGG	CCGAGTGAAGAACTGCACG	255
cg3282	ATGACCTGCGGACACTGC	TCAGGACAAGACGGTGTAG	180
*gntV*	TCCGTCGGTAAAGCCCTAGC	CGGTTCCTGGGCATTTGGTG	238
*proC*	CGCGGCCAACATGAATCCAC	GGCCATGCTGACCACAACAC	232
*proP*	TCGACTGGTGGTGAATATGC	GAATACGCCAACCGAAATCC	202
*pstC*	AATGCGAACTCCTCTCAGAC	AATCCGCCAATACCTTCAGC	206
*pstS*	TCCGCAATGGACTACTTTGG	AACTGGGCCGATAACGAATG	222


## Results

### *C. glutamicum* Can Use L-PA for Osmoprotection

**FIGURE 1:**
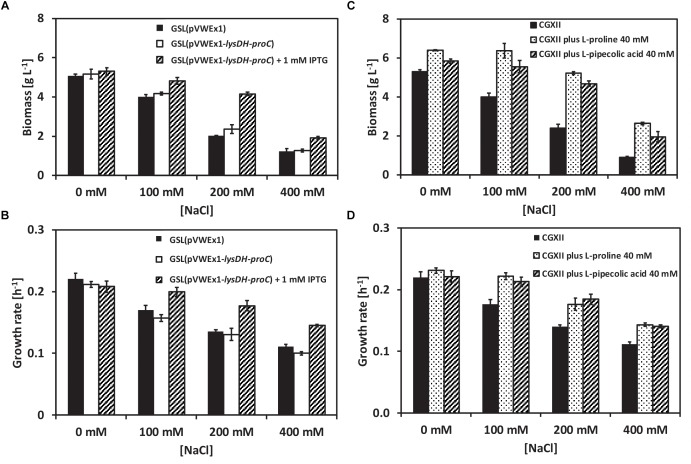
Growth of the *C. glutamicum* strains GSL(pVWEx1) and GSL(pVWEx1-*lysDH-proC*) under different osmotic conditions. **(A)** Biomass formation in g L^-1^ of GSL(pVWEx1) and GSL(pVWEx1-*lysDH-proC*) strains in glucose minimal medium supplemented with 0, 100, 200, or 400 mM of NaCl. **(B)** Growth rates values in h^-1^ of GSL(pVWEx1) and GSL(pVWEx1-*lysDH-proC*) strains in glucose minimal medium supplemented with 0, 100, 200, or 400 mM of NaCl. **(C)** Biomass formation in g L^-1^ of GSL(pVWEx1) strain in glucose minimal medium supplemented with 0, 100, 200, or 400 mM of NaCl with 40 mM of L-proline or 40 mM of L-PA or nothing. **(D)** Growth rates values in h^-1^ of GSL(pVWEx1) strain in glucose minimal medium supplemented with 0, 100, 200, or 400 mM of NaCl with 40 mM of L-proline or 40 mM of L-PA or nothing. Values represent means and standard deviations.

Structurally, L-PA is related to L-proline. To check whether L-PA functions as osmoprotectant in *C. glutamicum* the L-PA producer GSL(pVWEx1-*lysDH-proC*) (Pérez-García et al., 2016) was grown in glucose-minimal medium supplemented with 0, 100, 200, and 400 mM of NaCl using a BioLector system. Production of L -PA was induced by adding IPTG. When not induced for L-PA production, strains GSL(pVWEx1) and GSL(pVWEx1-*lysDH-proC*) showed decreasing growth rates (Figure 1A) and maximal biomass formation (Figure 1B) with increasing NaCl concentration. However, when L-PA production was induced, *C. glutamicum* GSL(pVWEx1-*lysDH-proC*) grew faster (Figure 1A) and to higher biomass concentrations (Figure 1B) in the presence of NaCl than the parent strain GSL(pVWEx1) (Figure 1). This indicated that biosynthesis of L-PA helps *C. glutamicum* to withstand hyperosmolar conditions.

To test if also exogenously added L-PA is beneficial for *C. glutamicum* when grown under hyperosmolar conditions, 40 mM of either L-PA or L-proline were added to the glucose-minimal medium and growth of *C. glutamicum* GSL(pVWEx1) was monitored (Figure 1C,D). The exogenous addition of L-PA or L-proline improved growth of strain GSL(pVWEx1) in the presence of all NaCl concentrations tested (Figure 1C,D). Thus, under the chosen conditions L-PA functions as well as the known osmoprotectant of *C. glutamicum*, L-proline, in promoting growth under hyperosmolar conditions. While several transport proteins for the compatible solute L-proline are known, transport of L-PA has not yet been studied.

### The Mechanosensitive Channel YggB May Play a Role as Escape Valve for L-PA

**FIGURE 2:**
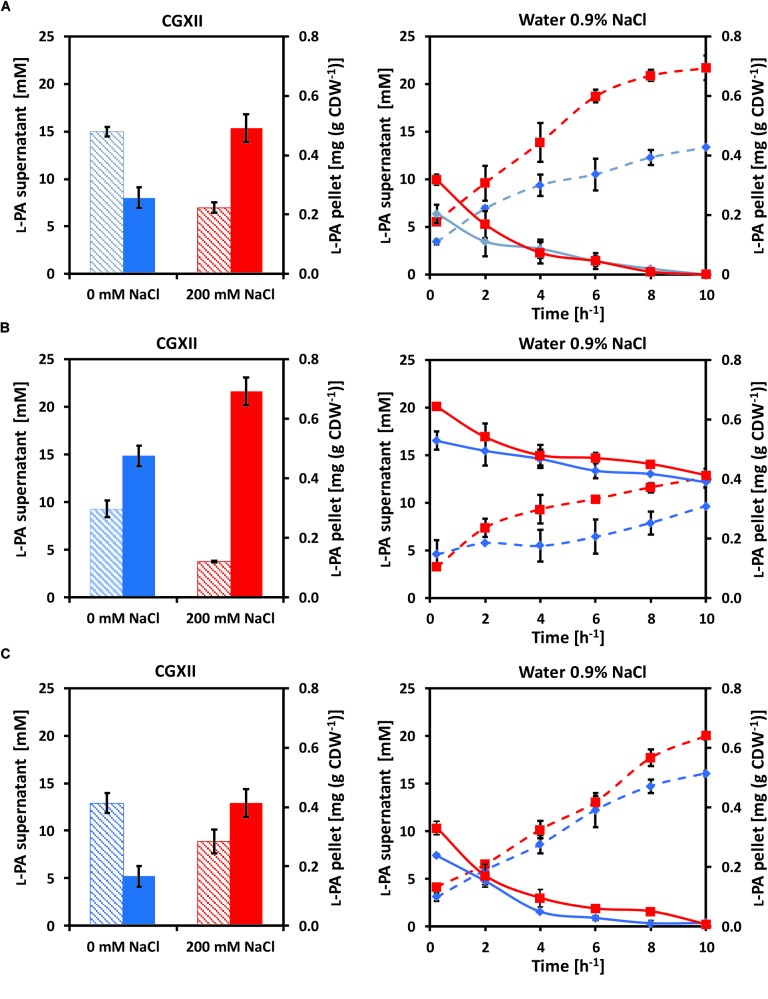
Intracellular (filled columns, straight lines) and extracellular (dashed columns, dashed lines) L-PA concentration profiles of the strains **(A)** GSL(pVWEx1-*lysDH-proC*), **(B)** GSLΔ*yggB*(pVWEx1-*lysDH-proC*) and **(C)** GSLΔ*yggB*(pVWEx1-*lysDH-proC*)(pEKEx3-*yggB*) after growth in glucose-minimal medium (left panels) without added NaCl (blue) or with 200 mM NaCl (red) and after osmotic downshift to 0.9% NaCl (right panels). Values represent means and standard deviations.

Osmo compatible solutes accumulate intracellularly under hyperosmolar conditions and are released from the cell upon osmotic downshift. Since YggB has been shown to be a key player in osmoregulation in *C. glutamicum* (Börngen et al., 2010), *yggB* deletion mutants were also analyzed. Here, the accumulation and release of L-PA by *C. glutamicum* cells were analyzed (Figure 2). *C. glutamicum* cells were cultivated in 50 mL glucose-minimal medium without (blue columns) or with (red columns) 200 mM NaCl. When glucose was depleted 1 mL supernatant and 2 mL pellet were collected to measure the extracellular (dashed columns) and intracellular (filled columns) concentrations of L-PA (Figure 2, left panels). The rest of the pellet of cells that grew in CGXII (blue lines) or CGXII + 200 mM NaCl (red lines) was transferred to 35 mL milliQ-water 0.9% NaCl to force an osmotic downshift while keeping the cells intact. The extracellular (dashed lines) and intracellular (solid lines) concentrations of L-PA were monitored over time (Figure 2, right panels). The experiment was performed with the L-PA producing strain GSL(pVWEx1-*lysDH-proC*) (Figure 2A); a *yggB* deletion mutant of this strain lacking the MSC YggB, GSLΔ*yggB*(pVWEx1-*lysDH-proC*) (Figure 2B); and a derived strain expressing *yggB* from a plasmid for complementation of the *yggB* deletion, GSLΔ*yggB*(pVWEx1-*lysDH-proC*)(pEKEx3-*yggB*) (Figure 2C). After growth in CGXII minimal medium with 200 mM NaCl, the intracellular L-PA concentrations were higher than after growth in CGXII minimal medium without added NaCl (Figure 2A), left). Upon osmotic downshift, L-PA was released from cells grown without NaCl and accumulated in the medium with a rate of 0.97 ± 0.04 mM h^-1^. However, when cells grown with 200 mM NaCl were subjected to osmotic downshift, L-PA was released with a 75% higher rate and to an about 1.6 higher concentration (Figure 2A, right and Table 4).

**Table 4:** Rates of decrease of intracellular L-PA concentrations and of L-PA accumulation in the cultivation medium upon osmotic downshock of different *C. glutamicum* strains after growth in glucose minimal medium with or without 200 mM NaCl.

	Rates of decrease of intracellular L-PA concentrations (μg g CDW^-1^ h^-1^)		Rates of L-PA accumulation in the supernatant (mM h^-1^)	
		
Strain	After growth with 0 mM NaCl	After growth with 200 mM NaCl	After growth with 0 mM NaCl	After growth with 200 mM NaCl
GSL(pVWEx1-*lysDH-proC*)	19.3 ± 0.2	30.2 ± 0.4	0.97 ± 0.04	1.73 ± 0.02
GSL**Δ*yggB***(pVWEx1-lysDH-proC)	14.0 ± 0.2	20.8 ± 0.3	0.47 ± 0.13	0.88 ± 0.17
GSL**Δ**yggB****(pVWEx1-*lysDH-proC*)(pEKEx3-***yggB***)	22.9 ± 0.3	28.9 ± 0.4	1.37 ± 0.16	1.68 ± 0.14


*Values represent means and standard deviations. Important strain features are depicted in bold.*

When growing the strain GSLΔ*yggB*(pVWEx1-*lysDH-proC*) on CGXII with or without 200 mM NaCl the internal accumulation of L-PA increased 80 and 40%, respectively, as compared with the strain GSL(pVWEx1-*lysDH-proC*) (Figure 2B, left). On 0.9% NaCl, the L-PA external accumulation rates decreased to the half (Figure 2B, right and Table 4). Therefore, the deletion of *yggB* slowed down L-PA export but was not enough to fully avoid it. The strain GSLΔ*yggB*(pVWEx1-*lysDH-proC*)(pEKEx3-*yggB*) showed similar L-PA internal/external profiles in both CGXII and water as compared with the strain GSL(pVWEx1-*lysDH-proC*) (Figure 2C, right and Table 4).

### Comparative RNAseq Analysis of a L-PA Producing *C. glutamicum* Strain, a L-Proline Producing Strain and the Respective Control Strains

Under the assumption that genes relevant for production of either L-PA or L-proline are differentially expressed when comparing L-PA producing *C. glutamicum* strain GSL(pVWEx1-*lysDH-proC*) with its parent strain GSL(pVWEx1) and of L-proline producing *C. glutamicum* strain JJ004 with its parent strain JJ001, comparative RNAseq analysis was performed. *C. glutamicum* strains GSL(pVWEx1-*lysDH-proC*), GSL(pVWEx1), JJ004 and JJ001 were grown in glucose minimal medium with 1 mM IPTG after inoculation with an initial OD600 of 1. Samples for RNA preparation were harvested after 8 h of inoculation at an OD600 of 7.6 ± 0.4 and 7.7 ± 0.4, respectively, for strains GSL(pVWEx1-*lysDH-proC*) and GSL(pVWEx1). Sample for strains JJ004 and JJ001 were harvested 6 h after inoculation at an OD600 of 7.6 ± 0.1 and 8.0 ± 0.2, respectively. RNA and library preparation, sequencing, read mapping and differential gene expression analysis using the statistical method DEseq (Naville et al., 2011) was performed as described in Section “Materials and Methods.” Full data is available as Gene Expression Omnibus GSE122249 data set at http://www.ncbi.nlm.nih.gov/geo/. As compared to the respective control strains, 5 genes showed increased RNA levels and 17 genes decreased RNA levels in the L-PA producing strain, while 26 genes showed increased RNA levels and 33 genes decreased RNA levels in the L-proline producing strain (Table 5).

**Table 5:** Comparative RNAseq analysis of L-proline producing strain JJ004, its isogenic non-producing control strain JJ001, L-PA producing *C. glutamicum* strain GSL(pVWEx1-*lysDH-proC*) and its isogenic non-producing control strain GSL(pVWEx1).

		Log_2_ fold change of RNA level (*P*-value < 0.01)	
		
Locus or gene	Product	JJ004/ control JJ001	GSL(pVWEx1-*lysDH-proC*)/ control GSL(pVWEx1)
cg0018	Conserved hypothetical membrane protein	0.8	-1.0
cg0107	Putative secreted protein	0.3	-1.0
cg0175	Putative secreted protein	-0.8	-1.2
cg0282	CsbD family protein involved in stress response	-0.8	-0.6
*ctpA*	Putative Cu^2+^ transporting P-type ATPase	1.1	0.2
***proC***	Pyrroline-5-carboxylate reductase	0.0	4.7
*glyR*	Transcriptional activator of *glyA*, ArsR-family	1.0	-0.5
**cg0569**	Putative Cd^2+^ transporting P-type ATPase	1.0	1.7
*whiB3*	Transcriptional regulator protein, WhiB-family	2.6	-0.5
*prpC2*	2-Methylcitrate synthase	0.9	0.7
cg0770	ABC-type putative iron-siderophore transporter, permease subunit	-1.9	0.3
*irp1*	ABC-type putative iron-siderophore transporter, substrate-binding lipoprotein	-2.3	-0.3
*whcE*	Transcriptional regulator, WhiB-family	0.8	-0.4
*pdxS*	Pyridoxal 5’-phosphate (PLP) synthase subunit S	-0.8	0.0
*pdxT*	Pyridoxal 5’-phosphate (PLP) synthase subunit T	-1.0	0.5
cg0924	ABC-type putative iron-siderophore transporter, substrate-binding lipoprotein	-1.7	-0.5
cg0926	ABC-type putative iron-siderophore transporter, permease subunit	-1.3	-0.2
cg0935	Conserved hypothetical protein	-0.4	-1.3
*rpf1*	RPF-protein precursor	0.9	-0.4
cg0952	Putative integral membrane protein	-0.9	-0.3
***betP***	Na^+^/glutamate symporter	-1.2	-0.7
cg1091	Hypothetical protein	-1.6	-1.0
cg1091	Hypothetical protein	-1.6	-1.0
cg1109	Hypothetical protein	-0.3	-1.1
cg1279	Putative secreted protein	0.8	0.6
cg1291	Putative membrane protein	1.2	-0.1
cg1293	Putative secreted protein	-0.8	-0.4
*putP*	Na^+^/proline symporter	-0.8	-0.1
cg1419	Putative secondary Na^+^/bile acid symporter, bile acid:Na^+^ symporter (BASS) family	-1.7	-0.2
*lysE*	L-Lysine efflux permease	-5.4	-0.5
*leuC*	3-Isopropylmalate dehydratase, large subunit	-0.8	0.0
*ptsG*	Phosphotransferase system (PTS), glucose-specific enzyme IIBCA component	-0.9	0.1
cg1604	Secreted protein, putative channel protein	-0.9	0.1
**cg1665**	Putative secreted protein	-1.7	-0.9
cg1746	Putative membrane protein	0.8	n.d.
cg1897	Putative secreted protein	-3.1	n.d.
cg1930	Putative secreted hydrolase	-1.1	n.d.
cg2068	Hypothetical protein	-1.0	n.d.
*psp1*	Putative secreted protein	-1.6	n.d.
*int2’*	Putative phage Integrase (N-terminal fragment)	-1.6	n.d.
cg2181	ABC-type putative dipeptide/oligopeptide transporter, substrate-binding lipoprotein	-0.8	0.1
*xerC*	Putative site-specific recombinase	-0.2	-1.2
cg2402	Secreted protein NLP/P60 family	0.8	-0.1
cg2425	Putative permease	0.7	0.3
cg2477	Conserved hypothetical protein	0.0	-0.8
cg2564	Conserved hypothetical protein	-1.7	-0.5
*catA*	Catechol 1,2-dioxygenase	0.3	0.7
cg2651	Conserved hypothetical protein, pseudogene	-0.6	-1.4
**cg2677**	ABC-type putative dipeptide/oligopeptide transporter, permease subunit	0.0	0.9
***gntV***	Gluconokinase	-0.5	1.8
*rpmJ*	50S ribosomal protein L36	-0.3	-1.0
*pstB*	ABC-type phosphate transporter, ATPase subunit	1.3	0.1
*pstA*	ABC-type phosphate transporter, permease subunit	1.2	0.5
***pstC***	ABC-type phosphate transporter, permease subunit	1.5	0.3
***pstS***	ABC-type phosphate transporter, substrate-binding lipoprotein	1.3	0.1
**cg2851**	Branched-chain amino acid aminotransferase, AT class III/4-amino-4-deoxychorismate lyase	0.0	-1.6
cg2875	Hypothetical protein	0.8	-0.3
cg2908	Putative membrane protein	-1.0	0.1
*pck*	Phosphoenolpyruvate carboxykinase (GTP)	0.8	0.0
*gntP*	Gluconate:H^+^ symporter	-1.0	0.1
cg3218	Pyruvate kinase-like protein	1.2	0.4
*ldh*	L-Lactate dehydrogenase, NAD-dependent	0.9	0.0
**cg3254**	Putative membrane protein	-0.8	-0.9
cg3271	SAM-dependent methyltransferase	0.3	-1.1
cg3281	Putative Cu^2+^ transporting P-type ATPase	1.2	-0.4
**cg3282**	Putative Cu^2+^ transporting P-type ATPase	1.4	0.0
cg3326	Hypothetical protein	1.1	0.8
*mez*	Malic enzyme	0.9	0.3
***proP***	Proline/betaine permease	-1.2	-1.2
cg3402	Putative Hg^2+^ permease, MerTP-family	1.2	0.0
cg3404	ABC-type putative iron(III) dicitrate transporter, substrate-binding lipoprotein	-2.1	-0.4
cg4014	Conserved hypothetical protein, possibly involved in stress response	0.3	-1.0
cg4019		0.8	0.1
cg4021		-0.6	-1.1


*Names of genes used for subsequent qRT-PCR analysis are given in bold. n.d., not detected.*

**FIGURE 3:**
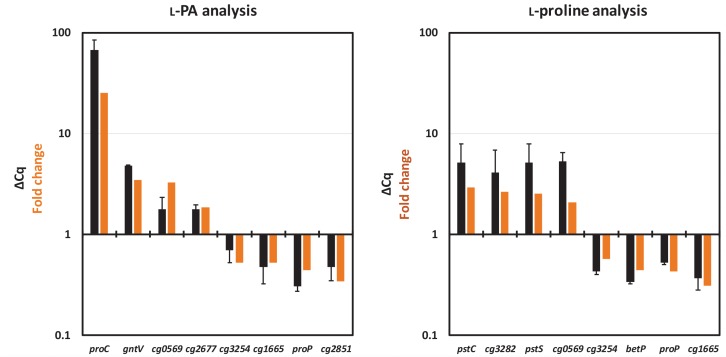
Comparison of relative gene expression values obtained by qRT-PCR analysis (black bars) with those obtained by RNAseq analysis (orange bars). RNAseq data from Table 5 and qRT-PCR data (ΔCq) collected for the L-PA analysis (**left**) and for the L-proline analysis (**right**) are listed. The values from the qRT-PCR are given as means and standard deviations.

The results obtained in the RNAseq analysis were validated by the analysis of gene expression patterns by qRT-PCR. For each analysis eight genes were selected, four upregulated and four downregulated genes. As shown in Figure 3, the relative gene expression levels obtained in qRT-PCR confirmed the pattern of their differential gene expression (fold change value) obtained in the RNAseq analysis.

In the L-proline producing strain JJ004, *pstSCAB* encoding phosphate ABC uptake system, genes for divalent metal transporter proteins (*ctpA*, cg0569, cg3281, cg3282, and cg3402), for transcriptional regulators (*glyR, whiB3*, and *whcE*) and for enzymes of central carbon metabolism (*pck, ldh* and *mez*) showed higher RNA levels than in the control strain JJ001 (Table 5). As compared to JJ001, RNA levels were lower in JJ004 for genes encoding iron-siderophore ABC uptake systems (cg0770, *irp1*, cg0924 and cg0926), the lysine/arginine permease gene *lysE* and genes for uptake of L-proline and other compatible solutes (*betP, putP* and *proP*) (Table 5). Thus, it appears that upon overproduction of L-proline, genes for its uptake from the culture medium are downregulated.

As expected, RNAseq analysis of the L-PA producing strain revealed increased expression of pyrroline-5-carboxylate reductase gene *proC* since it is expressed from plasmid pVWEx1-*lysDH-proC* (Table 5). Other genes showing increased RNA levels upon L-PA production were the divalent metal transporter protein gene cg0569, the gluconokinase gene *gntV* and the catechol 1,2-dioxygenase gene *catA* (Table 5). Genes showing decreased RNA levels upon L-PA production were the putative site-specific recombinase gene *xerC* and the compatible solute transport gene *proP* (Table 5). Notably, the genes *betP* and *putP* coding for uptake systems of L-proline and other compatible solutes did not show increased RNA levels. Thus, ProP was chosen as potential candidate for import of L-PA into the *C. glutamicum* cell.

### Role of the Carrier ProP During Growth With L-PA as Osmo Compatible Solute

**FIGURE 4:**
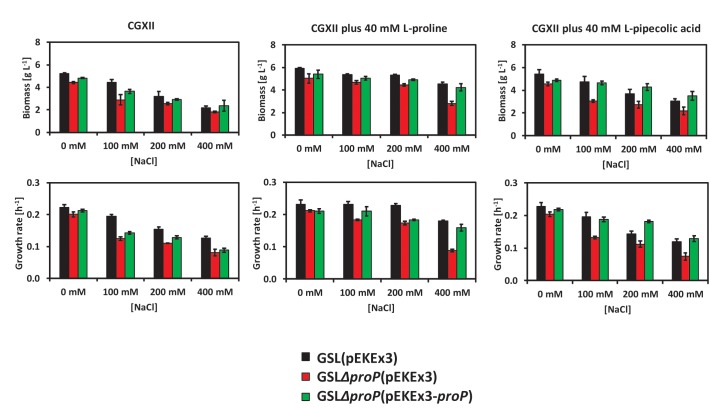
Growth behavior of the *C. glutamicum* strains GSL(pEKEx3) (black bars), GSLΔ*proP*(pEKEx3) (red bars) and GSLΔ*proP*(pEKEx3-*proP*) (green bars) under different osmotic conditions. Biomass formation in g L^-1^ (top-left) and growth rate in h^-1^ (down-left) when growing the strains in glucose minimal medium supplemented with 0, 100, 200, or 400 mM of NaCl. Biomass formation in g L^-1^ (top-middle) and growth rate in h^-1^ (down-middle) when growing the strains in glucose minimal medium supplemented with 0, 100, 200, or 400 mM of NaCl an in presence of 40 mM of L-proline. Biomass formation in g L^-1^ (top-right) and growth rate in h^-1^ (down-right) when growing the strains in glucose minimal medium supplemented with 0, 100, 200, or 400 mM of NaCl an in presence of 40 mM of L-PA. Values represent means and standard deviations.

Deduced from the RNAseq data, we speculated that the carrier ProP may play a role with regard to L-PA as osmo compatible solute of *C. glutamicum*. ProP is used by *C. glutamicum* as the main osmoregulated uptake system for L-proline (Peter et al., 1998). To test if the absence of ProP affects the use of *C. glutamicum* of L-PA under hyperosmolar conditions, strain GSLΔ*proP* was constructed. The strains GSL(pEKEx3) (Figure 4, black columns), GSLΔ*proP*(pEKEx3) (Figure 4, red columns) and GSLΔ*proP*(pEKEx3-*proP*) (Figure 4, green columns) were grown in glucose minimal medium supplemented with 0, 100, 200, or 400 mM of NaCl using a BioLector system. CGXII contains 200 mM MOPS buffer, thus, has a relatively high osmolarity: about 1,1 osmol/kg without added NaCl as compared to about 1,3 osmol/kg CGXII medium with 400 mM NaCl (Börngen et al., 2010). In addition, the effect of externally added L-proline or L-PA was tested. Data for final biomass formation and growth rate was collected for all conditions (Figure 4). It was observed that the strains carrying the deletion of *proP* suffered more from the hyperosmotic conditions as compared to the control *C. glutamicum* strain GSL(pEKEx3) or the complementation strain GSLΔ*proP*(pEKEx3-*proP*) (Figure 4). When 40 mM of L-proline was supplemented as osmo compatible solute to the minimal medium the growth rates and final biomass concentrations were reduced to a lesser extent than when 40 mM L-PA was added (Figure 4). Thus, ProP plays an important role when L-proline and L-PA are used as osmo compatible solutes in *C. glutamicum*. These findings are commensurate with the view that ProP does not only import L-proline into the *C. glutamicum* cell, but also L-PA. However, future in depth biochemical analysis of L-PA uptake are needed to determine the respective kinetic parameters of L-PA uptake.

## Discussion

In this study, L-PA was shown to be an osmo compatible solute for *C. glutamicum.* L-PA cannot be synthesized by *C. glutamicum* wild type, but can be imported from the environment. The synthesis and/or accumulation of compatible solutes is a widespread microbial strategy against osmolarity fluctuations (da Costa et al., 1998; Kempf and Bremer, 1998; Wood et al., 2001; Czech et al., 2018). High cytoplasmic concentrations of compatible solutes also stabilize protein folding and ribosomes and protect the DNA, increasing the resistance to other types of stress such as high and low temperatures and radiation (Li and Gänzle, 2016; Sajjad et al., 2018; Tribelli and López, 2018). The osmoprotection mechanisms for the microbial cell factories *E. coli, Bacillus subtilis* and *C. glutamicum* are described (Kempf and Bremer, 1998; Wood et al., 2001; Morbach and Krämer, 2003; Hoffmann and Bremer, 2017). These and other non-halophilic bacteria accumulate K ions, Na ions or glutamate after an osmotic upshock as first response before these ions are exchanged against compatible solutes either by synthesis or uptake (Wood, 1999). *C. glutamicum* either synthesizes glutamine, proline or trehalose after an osmotic upshift or imports glycine betaine, proline or ectoine (Frings et al., 1993; Guillouet and Engasser, 1995; Skjerdal et al., 1996). In *C. glutamicum*, biosynthesis of proline involves one set of genes (*proA* for g-glutamyl phosphate reductase, *proB* for g-glutamyl kinase and *proC* for pyrroline 5-carboxylate reductase), while *B. subtilis* possesses one set of proline biosynthesis genes for anabolic purposes and a second osmostress-induced set (Bremer, 2000). Similarly, biosynthesis and internal content of L-PA is regulated by external osmolality in *Brevibacterium ammoniagenes* (Gouesbet et al., 1992). It has been described that *C. glutamicum* prefers uptake of compatible solutes to their synthesis because uptake of compatible solutes is faster and energetically more favorable than their synthesis (Morbach and Krämer, 2003). In *C. glutamicum*, glycine betaine is the most effective osmoprotectant among those that can be imported from the environment, followed by ectoine and proline (Farwick et al., 1995). Here, we have shown that 40 mM L-PA are almost as effective as 40 mM proline for osmoprotection of a lysine producing *C. glutamicum* strain (Figure 1, 4). The improved growth in the presence of increasing salt concentrations is not due to catabolism of L-PA since *C. glutamicum* can use L-PA neither as carbon source nor as nitrogen source (Pérez-García et al., 2016). Rather, the external addition of L-PA is advantageous since resources for biosynthesis of L-proline for osmoprotection are not required. An osmoprotective role of L-PA has also been shown for *E. coli* since the addition of 1 mM of DL-PA decreased the inhibitory growth effect of 200–700 mM NaCl in 0.2% glucose minimal medium (Gouesbet et al., 1994). When growing *S. pomeroyi* in a mineral salts medium containing 400 mM NaCl, the supplementation of 5–20 mM of L-PA improved the growth of the microorganism (Neshich et al., 2013). In *Sinorhizobium meliloti*, both isomers, L-PA and D-PA have to be added for osmoprotective activity (Gouffi et al., 2000). Previously, we have developed strains for sustainable production of L-PA (Pérez-García et al., 2016, 2017a). Here, we have shown that recombinant *C. glutamicum* engineered for L-PA overproduction showed improved growth characteristics under hyperosmolar conditions (Figure 4). Thus, L-PA functions as osmo compatible solute not only when imported from the environment, but also when synthesized *de novo*. In these recombinant *C. glutamicum* strain synthesis is not osmostress-induced as described for certain plants. For example, in rapeseed leaf tissues, L-PA synthesis from L-lysine via the lysine-ketoglutarate reductase/saccharopine dehydrogenase pathway is osmo-dependent (Moulin et al., 2006).

Although uptake of osmo compatible solutes is important and energetically favored over their *de novo* synthesis, a *C. glutamicum* mutant devoid of the five known uptake systems for compatible solutes survived under hyperosmolar conditions (Steger et al., 2004) which has been attributed to *de novo* synthesis of proline, glutamine, and trehalose (Rönsch et al., 2003). The secondary carriers PutP, BetP, EctP, LcoP, and ProP differ by their substrate spectrum and substrate affinities, however, they show a degree of substrate redundancy that is typical for soil bacteria (Peter et al., 1997, 1998; Wood et al., 2001; Weinand et al., 2007). BetP is a high affinity carrier specific for betaine (Peter et al., 1997). EctP is a low affinity carrier for betaine, ectoine and proline and LcoP a low affinity carrier for betaine and ectoine, whereas the carrier ProP shows high affinity for its substrates proline and ectoine (Peter et al., 1998; Steger et al., 2004). PutP imports proline with high affinity for anabolic purposes (Peter et al., 1997). Based on differential gene expression analysis ProP was identified as possible L-PA import system in this work (Table 5). In *E. coli* various structural analogs of L-proline such as azetidine-2-carboxylate, L-pipecolic acid or 5-hydroxy-L-pipecolic acid enter the cell through ProP or ProU transport systems (Gouesbet et al., 1994). The *E. coli* proline/glycine betaine transporter ProP shares 36.8% identical amino acids with ProP from *C. glutamicum.* Here, growth analysis of *C. glutamicum* mutants lacking *proP* revealed perturbed growth under hyperosmolar conditions in the absence of *proP* (Figure 4). Since mutants lacking *proP* still possess functional EctP and PutP these carriers apparently do not contribute to uptake of L-PA under the chosen conditions, and, thus, ProP may act as major L-PA import system in *C. glutamicum*. Detailed biochemical transport assays will have to be performed in the future to characterize L-PA uptake by ProP.

It was also shown in this work that the MSC YggB performs as a major escape valve for L-PA in *C. glutamicum* (Figure 2). After an osmotic downshift compatible solutes are released to the medium involving MSCs (Morbach and Krämer, 2003). In particular, the MSC YggB was described as the main export system of L-glutamate in *C. glutamicum* (Nakamura et al., 2007). In *C. glutamicum* it is known that the use of biotin limitation, penicillin treatments or surfactants alter membrane tension by inhibiting lipid or peptidoglycan synthesis which triggers conformational changes in YggB allowing L-glutamate export (Duperray et al., 1992; Gutmann et al., 1992). In addition, betaine efflux induced by osmotic downshock was reduced upon deletion of *yggB* (Nottebrock et al., 2003). Thus, the decreased rate of L-PA accumulation in the supernatant as consequence of *yggB* deletion suggests that L-PA may be exported from the *C. glutamicum* cell by YggB. In depth biochemical analysis is required to characterize export of L-PA by YggB.

Due to their diverse applications in drug development, food industry, skin care products and cosmetics (Graf et al., 2008; Jorge C.D. et al., 2016; Li and Gänzle, 2016) the biotechnological production of compatible solutes has gained increasing momentum recently (Sauer and Galinski, 1998; Jensen and Wendisch, 2013; Tan et al., 2016; Chen et al., 2017). This included the establishment of strains that produce and secrete compatible solutes such as ectoine, L-PA or α-D-glucosylglycerol that are not synthesized by the wild-type strains (Ning et al., 2016; Pérez-García et al., 2017a,b; Ying et al., 2017; Roenneke et al., 2018). Production of L-PA by recombinant *E. coli* expressing the gene for lysine cyclodeaminase from *Streptomyces hygroscopicus* was established with a titer of 5.33 g L^-1^ L-PA and a yield of 0.13 g L^-1^ of glucose obtained in fed-batch cultivation and a titer of 0.64 g L^-1^ L-PA in shake flasks (Ying et al., 2017). Our previous work on establishing L-PA production in *C. glutamicum* led to superior values: 14.4 g L^-1^ L-PA and a yield of 0.20 g g^-1^ in fed-batch cultivation and a titer of 3.9 g L^-1^ L-PA in shake flasks (Pérez-García et al., 2017a). Although *de novo* synthesized L-PA protected *C. glutamicum* against high salt conditions, we have observed that in the presence of 200 mM NaCl the L-PA titer in the supernatant was reduced from about 15 mM (about 1.9 g L^-1^) to about half (Figure 2A, left panel). Thus, hyperosmolar conditions are not favorable for L-PA production by the *C. glutamicum* recombinant strains described here. However, while less L-PA was secreted under hyperosmolar conditions, more L-PA accumulated intracellularly (Figure 2A, left panel). After osmotic downshift, L-PA was released to the culture medium accumulating to about 22 mM (about 2.8 g L^-1^) (Figure 2A, right panel). Therefore, in principle, the described *C. glutamicum* strains could be used in a process called “bacterial milking” (Sauer and Galinski, 1998). The Gram-negative bacterium *Halomonas elongata* was grown to high-cell-density (48 g cell dry weight per liter) before being exposed to alternating hyper- and hypo-osmolar conditions. Ectoine released to the hypo-osmolar medium was harvested by crossflow filtration and by this procedure 0.16 g of ectoine per cycle per gram cell dry weight could be produced (Sauer and Galinski, 1998). As use of high-salinity media in fermentation processes is costly and poses challenges with regard to the design and durability of bioreactors, it is generally assumed that direct fermentative production is preferred over the “bacterial milking” process. To determine if this notion holds true for the L-PA producing *C. glutamicum* strains described here, a head-to-head comparison of these strains operated in a fed-batch fermentation process vs. a “bacterial milking” process will have to be performed after each process has been thoroughly optimized by process intensification. In addition, strain optimization by transport engineering, as described for the production of amino acids (Nakamura et al., 2007; Blombach et al., 2009), non-proteinogenic amino acids (Jorge J.M.P. et al., 2016; Pérez-García et al., 2017a), diamines (Kind et al., 2014; Nguyen et al., 2015) or organic acids (Huhn et al., 2011), may be required.

## Data Availability

The datasets generated for this study can be found in http://www.ncbi.nlm.nih.gov/geo/, GSE122249.

## Author Contributions

FP-G, LB, and VW designed the study. FP-G and LB performed the experiments. FP-G, LB, and VW analyzed the data. FP-G and LB drafted the manuscript. VW finalized the manuscript. All authors read and approved the final version of the manuscript.

## Conflict of Interest Statement

The authors declare that the research was conducted in the absence of any commercial or financial relationships that could be construed as a potential conflict of interest.
